# Environmental gradients in old‐growth Appalachian forest predict fine‐scale distribution, co‐occurrence, and density of woodland salamanders

**DOI:** 10.1002/ece3.4736

**Published:** 2018-12-04

**Authors:** J. Alex Baecher, Stephen C. Richter

**Affiliations:** ^1^ Department of Biological Sciences and Division of Natural Areas Eastern Kentucky University Richmond Kentucky

**Keywords:** Amphibians, forest canopy structure, natural disturbance regimes, *Plethodon kentucki*, *Plethodon richmondi*, population dynamics

## Abstract

Woodland salamanders are among the most abundant vertebrate animals in temperate deciduous forests of eastern North America. Because of their abundance, woodland salamanders are responsible for the transformation of nutrients and translocation of energy between highly disparate levels of trophic organization: detrital food webs and high‐order predators. However, the spatial extent of woodland salamanders’ role in the ecosystem is likely contingent upon the distribution of their biomass throughout the forest. We sought to determine if natural environmental gradients influence the fine‐scale distribution and density of Southern Ravine Salamanders (*Plethodon richmondi*) and Cumberland Plateau Salamanders (*P. kentucki*). We addressed this objective by constructing occupancy, co‐occurrence, and abundance models from temporally replicated surveys within an old‐growth forest in the Cumberland Plateau region of Kentucky. We found that *Plethodon richmondi* had a more restricted fine‐scale distribution than *P. kentucki* (mean occupancy probability [$\hat{\overset{¯}{\psi }}$] = 0.737) and exhibited variable density, from <250 to >1000 individuals per hectare, associated with increased soil moisture and reduced solar exposure due to slope face. While more ubiquitously distributed ($\hat{\overset{¯}{\psi }}$ = 0.95), *P. kentucki* density varied from <400 to >1,000 individuals per hectare and was inversely related to increased solar exposure from canopy disturbance and landscape convexity. Our data suggest co‐occurrence patterns of *P. richmondi* and *P. kentucki* are influenced primarily by abiotic conditions within the forest, and that populations likely occur independently and without evidence of biotic interaction. Given the critical role that woodland salamanders play in the maintenance of forest health, regions that support large populations of woodland salamanders, such as those highlighted in this study—mesic forest stands on north‐to‐east facing slopes with dense canopy and abundant natural cover, may provide enhanced ecosystem services and support the stability of the total forest.

## 1 INTRODUCTION

Analyzing the distribution and abundance of species along environmental gradients yields invaluable information about their niche requirements (Costa, Wolfe, Shepard, Caldwell, & Vitt, 2008), population dynamics (Peterman & Semlitsch, 2013), and biotic interactions (Maestre et al., 2010) and can even inform decisions about the management and restoration of landscapes for species conservation (Peterson, 2006). In unaltered landscapes, the distribution of species is a function of natural environmental gradients, which include abiotic factors (e.g., surface temperature, moisture, topographic relief, water and soil chemistry, and solar radiation) and biotic factors (e.g., vegetative structure and the presence of predators, prey, and mates; Van der Putten, Macel, & Visser, 2010). Taxa likely to exhibit strong responses to such natural gradients are those with limited dispersal capabilities (Cushman, 2006), low reproductive success (Elton, 1958), and acute sensitivity to environmental conditions (Buckley & Jetz, 2007).

One such group, amphibians, is particularly responsive to environmental gradients (Araújo et al., 2007; Semlitsch, Peterman, Anderson, Drake, & Ousterhout, 2015; Werner, Skelly, Relyea, & Yurewicz, 2007). Because of their highly permeable skin, amphibians are acutely sensitive to the chemical environment (Boone, Semlitsch, Little, & Doyle, 2007; Willson, Hopkins, Bergeron, & Todd, 2012), thermal and hydrologic regimes (Semlitsch et al., 2015; Walls, Barachivich, & Brown, 2013), and the microbiome (i.e., emerging pathogenic diseases; Carey et al., 2003; Collins et al., 2003). Amphibian population dynamics are closely tied to landscape structure (Hecnar & M'Closkey, 1996; Rothermel & Semlitsch, 2002) and prey availability (Greene, Lowe, & Likens, 2008), making them especially sensitive to habitat destruction and degradation (Brooks et al., 2002). These characteristics likely explain why amphibians are currently experiencing unprecedented and precipitous population declines on a global scale (Alford, Dixon, & Pechmann, 2001; Houlahan, Findlay, Benedikt, Meyer, & Kuzim, 2000; Stuart et al., 2004). Nevertheless, amphibians’ hypersensitivity to environmental conditions translates into an effective taxonomic indicator of ecosystem integrity (Welsh & Droege, 2001; Welsh & Ollivier, 1998).

Despite this sensitivity, amphibians represent a major component of biomass in aquatic (Gibbons et al., 2006), terrestrial (Burton & Likens, 1975b; Petranka & Murray, 2001), and riparian (Peterman, Crawford, & Semlitsch, 2008) ecosystems. Because the life history of many amphibians involves movement between and among aquatic and terrestrial ecosystems (Regester, Whiles, & Taylor, 2006), they are responsible for the transformation (Burton & Likens, 1975a) and translocation (Capps, Berven, & Tiegs, 2014; Luhring, DeLong, & Semlitsch, 2017) of substantial quantities of energy throughout the landscape. However, the role of energy transformation is not unique to biphasic organisms. Terrestrial woodland salamanders (Caudata: Plethodontidae: *Plethodon*), which lack aquatic larval stages (i.e., have direct development), are among the most abundant vertebrate animals in eastern deciduous forests of North America (Petranka & Murray, 2001; Semlitsch, O'Donnell, & Thompson, 2014), reaching densities between 0.73 and 18.46 individuals per m^2^ (O'Donnell & Semlitsch, 2015; Semlitsch et al., 2014). They also act as predators of detrital food webs (Best & Welsh, 2014; Davic & Welsh, 2004; Hutton, Price, & Richter, 2017) and represent a prey resource for a wealth of vertebrate and invertebrate predators (for a taxonomic review of *Plethodon* predators, see Semlitsch et al., 2014). As such, woodland salamanders are hypothesized to serve as a key energetic intermediary between highly disparate levels of trophic organization in terrestrial ecosystems (detrital communities and high‐order vertebrate predators; Burton & Likens, 1975b) and exert a significant, top‐down, regulatory force upon detrital food webs, leaf litter decomposition, and organic material retention (Burton & Likens, 1975a; Hairston, 1987). Therefore, woodland salamanders may significantly influence the direction and magnitude of energy flow through ecosystems (Davic & Welsh, 2004).

Wyman (1998) suggested that, through predation of detrital food webs, woodland salamanders (*Plethodon cinereus*, eastern red‐backed salamander) can indirectly reduce leaf litter processing rates, aiding in the retention of organic carbon in forests. However, additional studies have found that the significance, strength, and direction of top‐down effects on leaf litter decomposition and detrital communities is subject to variation (Best & Welsh, 2014; Hocking & Babbitt, 2014; Homyack, Sucre, Haas, & Fox, 2010; Walton, 2005; Walton & Steckler, 2005; Walton, Tsatiris, & Rivera‐Sostre, 2006). Recent evidence suggests that variation in the effects of woodland salamanders on forest floor dynamics is likely correlated with spatiotemporal variability in environmental conditions (Walton, 2013) and the abundance of salamander predators (Hickerson, Anthony, & Walton, 2017). Therefore, the nature of woodland salamanders’ role in terrestrial ecosystem nutrient cycling is likely contingent upon the spatial distribution of their biomass within the ecosystem (Hickerson et al., 2017; Semlitsch et al., 2014), which is influenced by spatial patterns in environmental conditions and resource availability (Milanovich & Peterman, 2016; Peterman & Semlitsch, 2013; Walton, 2013).

Numerous studies have found the distribution of woodland salamanders to be influenced chiefly by terrestrial ecosystem features such as soil moisture (Jaeger, 1971a; Peterman & Semlitsch, 2013; Wyman, 1988), availability of natural cover (i.e., coarse woody debris, rocky cover, and leaf litter; McKenny, Keeton, & Donovan, 2006; O'Donnell, Thompson, & Semlitsch, 2014), and forest composition/canopy structure (Gibbs, 1998; Peterman & Semlitsch, 2013). Furthermore, the presence of heterospecifics has been found to influence microhabitat usage (Farallo & Miles, 2016; Keen, 1982), distribution (Hairston, 1950; Jaeger, 1970, 1971a, b), and abundance (Hairston, 1951) of individual species. Thus, the species‐specific contribution of woodland salamanders to terrestrial ecosystem processes may be modified through population‐level effects of interspecific competition. Due to the diversity and endemism of woodland salamanders, particularly in Appalachian forests where their diversity is greatest (Dodd, 2004), community structure varies dramatically across physiographic regions. Therefore, community interactions are likely geographically nuanced and not easily generalizable from any single region.

Studies of the spatial population dynamics of woodland salamander species occurring in syntopy are needed to further understand the role of these animals in terrestrial ecosystems. Furthermore, woodland salamander populations in lower elevation Appalachian forests, like those of central Appalachia, have not been studied as thoroughly as in regions with greater topographic relief and higher proportions of land allocated for conservation. This study examines the population dynamics of Southern Ravine Salamander (*Plethodon richmondi*) and Cumberland Plateau Salamander (*P. kentucki*) within an old‐growth forest in the Cumberland Plateau region of Appalachia. In old‐growth forests of this region, variation in environmental conditions of the forest floor (e.g., soil moisture, availability of woody debris, and solar exposure) is largely influenced by canopy dynamics (Runkle, 1982). Tree mortality supplies woody debris to the forest floor (Harmon et al., 1986) and provides habitat for woodland salamanders (McKenny et al., 2006; Petranka, Brannon, Hopey, & Smith, 1994); however, resultant canopy gaps increase solar exposure, accelerating evapotranspiration. The size and persistence of canopy gaps represent a natural disturbance regime, which greatly modifies local environmental conditions (Schaetzl, Johnson, Burns, & Small, 1989; Scharenbroch & Bockheim, 2007). The objectives of this study were to determine if environmental gradients associated with the natural disturbance regime of an Appalachian old‐growth forest influence the fine‐scale distribution and density of *P. richmondi* and *P. kentucki*. Furthermore, this study sought to determine if patterns of salamander co‐occurrence vary along natural environmental gradients, and if those patterns are modified behaviorally through interspecific competition and territoriality. These objectives are addressed by constructing hierarchical models, which incorporate imperfect detection from temporally replicated surveys within an old‐growth forest.

## 2 MATERIALS AND METHODS

### 2.1 Study site

This study was conducted at Lilley Cornett Woods Appalachian Ecological Research Station (LCW), which contains 102 ha of old‐growth forest. Lilley Cornett Woods is a stable mixed mesophytic forest in the Cumberland Plateau region of southeastern Kentucky. With no history of timber harvest, the old‐growth forest at LCW has experienced no substantial anthropogenic disturbance with the exception of understory livestock grazing, which ended in the 1950s (Martin, 1975). Canopy disturbances in LCW are primarily stochastic (Davis, Chapman, Wu, & McEwan, 2015), and therefore, the distribution of canopy gaps is predicted to be uniform and resultant from endogenous processes. Of the three tracts of old‐growth forest at LCW, one tract, “Shop Hollow,” currently experiences little disturbance from human recreation (only guided hiking on a narrow, established trail) and features minimal understory vegetation. Shop Hollow features 57 permanent circular sample plots with a diameter of 32 m (800 m^2^), which were originally established by Martin (1975). Sample plots were stratified by elevation (lower [<345 m], middle [345–410 m], upper [411–467 m], and ridge [>467 m]). Data collection occurred at all sample plots free of intersecting streams (*N* = 40).

### 2.2 Amphibian sampling

This study relied upon visual encounter surveys (VES) to detect species, and therefore, all observations resulted from hand captures during standardized searching.

Each of the *N* = 40 sample plots was visited four times between 15 October 2016 and 13 November 2016 from 0800–2000 EST, with no less than five days occurring between visits. Locally, observations of *Plethodon* salamander activity in the fall season can rival, if not exceed, those of the spring (Baecher & Richter pers. obs., MacGregor pers. comm.), and therefore, this sample period was chosen as the most representative of true patterns in occurrence and abundance. VES were conducted along a linear 3‐m × 32‐m transect (96 m^2^), which intersected the center of each 800‐m^2^ circular sample plot at a randomly chosen bearing between 0° and 180°. The bearing of each transect was also randomized during each sequential visit, making the likelihood of sampling the same microhabitat at a given sample plot negligible. In LCW, woodland salamanders are found primarily by searching under natural cover (coarse woody debris [CWD] and rocks) on the forest floor. During VES, all CWD and rocky cover within the 96‐m^2^ transects were flipped, and microhabitats beneath were examined for the presence of salamanders before replacing cover items to their exact position.

### 2.3 Site covariates

During each visit, soil moisture was measured with a Pro Check moisture probe (Decagon Devices, Inc.) at five equidistant points along the transect within each sample plot, and therefore, estimates were obtained by averaging across transect (*N* = 5) and visit (*N* = 4). Quantification of forest canopy openness was achieved using hemispherical canopy photography (Baldwin, Calhoun, & DeMaynadier, 2006; Frazer, Lertzman, & Trofymow, 1997; Herbert, 1987). A single photograph of canopy structure was captured prior to leaf off with a 24‐megapixel digital single‐lens reflex camera (Nikon D7100) on automatic settings, fitted with a 180° lens (Nikon AF DX Fisheye‐Nikkor 10.5 mm f/2.8G ED; Nikon Instruments, Melville, NY, USA). Percent canopy openness was calculated by converting images into binary color (black pixels = closed canopy, white pixels = open canopy) using a binarization algorithm provided by the Auto Threshold Plugin for ImageJ software (Abramoff, Magalhaes, & Ram, 2004; Rasband, 2014), and then calculating the percent of white pixels in each frame.

A 1.11‐m^2^ digital elevation model was used to derive the following layers: aspect, slope, Topographic Position Index, and direct solar radiation. Aspect was scaled into a linear variable ranging from 0 (xeric, southwest‐facing slopes) to 2 (mesic, northeast‐facing slopes) using the Beers transformation (Beers, Dress, & Wensel, 1966; O'Donnell, Thompson, & Semlitsch, 2015). Topographic Position Index (TPI) is a measure of landscape convexity which was calculated by comparing the slope position of individual sample plots relative to a 150‐m^2^ surrounding landscape area using a neighborhood function (Guisan, Weiss, & Weiss, 1999). During the calculation of TPI, a suite of additional neighborhood scales was considered, beginning with a circular area of 50 m^2 ^and increasing incrementally by 50–1,500 m^2^. The most appropriate TPI scale was selected by correlating each TPI calculation with plot averages of raw salamander counts and identifying the scale with the highest correlation coefficient. Direct Solar Radiation—a component of the total solar radiation—represents the quantity of solar radiation remaining after a fraction is absorbed by the atmosphere (diffuse solar radiation) or reflected off of the earth's surface (reflected solar radiation). Normalized Difference Vegetation Index (NDVI) is a measure of vegetative cover (range: −1.0 [barren] to 1.0 [heavily vegetated]) and was derived using imagery from the 2016 National Agriculture Imagery Program. All data were gathered with ArcGIS 10.3 (ESRI, 2011). See Table 1 for a description of all sampling and site covariates.

**Table 1:** Description and summary statistics of covariates used in hierarchical models of Southern Ravine Salamander (*Plethodon richmondi*) and Cumberland Plateau Salamander (*P. kentucki*) occupancy and abundance

Parameter	Abbr.	Covariate description	Unit	Mean	Interquartile range	
Sampling						
Conditional capture probability, p_ψ_; effective detection probability, p_λ_	CWD	Abundance of coarse woody debris^a^	qty.	3	1	4
	DAY	Day of the year survey occurred	Julian date	306.50	300.80	312.20
	LLD	Leaf litter depth^a^	cm	5.45	4.45	6.75
	LUX	Luminous flux^a^	lumen/m^2^	510.40	175.00	740.00
	ROC	Abundance of rocky cover^a^	qty.	5	1	7
	TOD	Time of day survey occurred	24 hr time	1,310	1,102	1518
Site						
Occupancy probability, *ψ*; estimated abundance, *λ*	ASP	Beers‐transformed aspect^b^	range (0,2)	1.19	0.33	1.87
	CAN	Canopy openness^a^	%	26	20	28
	ELV	Elevation^b^	m	407.40	375.40	436.40
	MST	Soil moisture^a^	%	14	11	17
	RAD	Direct Solar Radiation^b^	Kilowatts hr^−1^ m^−2^	746.12	677.92	846.91
	TPI	Topographic Position Index^b^	c	14.20	4.05	23.55
	VEG	Normalized Difference Vegetation Index^b^	d	126.60	123.80	130.40

Note

Data collected in 2016 at Lilley Cornett Woods Appalachian Ecological Research Station (Letcher Co., Kentucky, USA). Covariates quantify two processes: “sampling” (detectability parameters) and “site” (species occupancy and abundance).

a Collected in situ.

b Spatially derived metric.

c Measure of slope position relative to surroundings (“+”=ridges, “−” = valleys, “0” = flat).

d Measure of vegetative density (−1.0 = barren, 1.0 = heavily vegetated).

### 2.4 Sampling covariates

The quantity of fallen coarse woody debris larger than 20 cm in diameter (Muller & Liu, 1991) and rocky cover within each VES transect were counted. Leaf litter depth was measured with a metric ruler at five equidistant points within each survey transect. Solar conditions during surveys were quantified by measuring the ambient luminous flux (perceived power of light) at breast height with a digital illuminance light meter (TekPower, model: LX1330B). Finally, date and time of day of each survey were recorded. See Table 1 for a description of all sampling covariates.

### 2.5 Occupancy, co‐occurrence, and abundance modeling

Because detection probabilities of salamanders were assumed <1, hierarchical models were used to approximate woodland salamander distributions and density from repeated surveys of unmarked animals (MacKenzie & Royle, 2005). Occupancy models (MacKenzie, Nichols, Hines, Knutson, & Franklin, 2002) were used to estimate the probability that a species occupied a given site (*ψ*), while N‐mixture models (Royle, 2004) were used to estimate species true population size (*λ*). Fitting occupancy and N‐mixture models followed a stepwise procedure: (a) models were constructed to estimate detection parameters, p, by holding the state parameters, occupancy and abundance, constant; (b) the model‐averaged estimated effect size ($\hat{\overset{¯}{\beta }}$) of each detection covariate was calculated using multi‐model inference (Burnham & Anderson, 2002; Mazerolle, 2006) to determine importance; (c) models were then constructed to estimate occupancy and abundance using covariates of detection selected from the previous step; (d) from the resulting models, $\hat{\overset{¯}{\beta }}$ was calculated for each site covariate to determine which was important in explaining occupancy and abundance; and (e) multi‐model inference was used to make predictions across all models. See Appendix S1–S5 for a complete list of models fitted. For examples of studies using similar stepwise procedures, see Govindan, Kéry, and Swihart (2012), Scherer, Muths, and Noon (2012), Kéry Guillera‐Arroita and Lahoz‐Monfort (2013), Peterman, Crawford, and Kuhns (2013), Peterman and Semlitsch (2013), and Jachowski, Millspaugh, and Hopkins (2016).

Prior to statistical analyses, all site and sampling covariates were standardized to a mean of zero and unit variance by subtracting the arithmetic mean and dividing by the standard deviation (as recommended by Fiske & Chandler, 2011, 2017). Models were fitted using a maximum‐likelihood approach with package “unmarked” (Fiske & Chandler, 2011) in the R programing environment (v. 3.4.1; R Core Team, 2017). Goodness‐of‐fit tests with 10,000 parametric bootstrap iterations were performed on the most highly parameterized (global) occupancy and N‐mixture models of each species and confirmed that empirical distributions did not significantly deviate from the theoretical distributions (occupancy: zero‐inflated binomial; N‐mixture: Poisson) used in each model (*p* > 0.05, $\hat{c}\approx 1$; Kéry & Royle, 2016). Goodness‐of‐fit tests and multi‐model inference to obtain predictions of occupancy, abundance, and detection were performed using R package “AICcmodavg” (Mazerolle, 2017).

Although occupancy and N‐mixture models both require an estimate of detectability to compute state parameters, the specific components of detection used by each are different (O'Donnell & Semlitsch, 2015). Most single‐season occupancy models, including the model used in this study, estimate the “conditional capture probability” (${\hat{p}}_{\psi }$), defined as the probability of capture, given the individual is present (capture probability|availability). For these terms, availability is defined as 1—(temporary emigration). Single‐season N‐mixture models estimate a form of detection which combines a term for the ability of the observer to capture an individual that is present (conditional capture probability) with a term for the individual's availability for capture (expressed as: availability × conditional capture probability) and is thus referred to as an “effective detection probability” (p*_λ_*). By exploiting the relationship between effective detection probability and conditional capture probability, estimates of population capture availability and temporary emigration (probability an animal is alive, but unavailable for capture) can be obtained from single‐season models mathematically.

Two‐species single‐season occupancy models (MacKenzie, Bailey, & Nichols, 2004) were used to estimate the probability that *P. richmondi* occupies a site or sites wherein *P*. *kentucki* is known to be present. Under the null hypothesis, the pattern and frequency of co‐occurrence does not vary across environmental gradients. This hypothesis was tested by comparing a null model of co‐occurrence, wherein the pattern in which species co‐occur at sites is unrelated to environmental conditions (essentially random), to models of co‐occurrence, which predict co‐occurrence patterns relating to environmental gradients. Using the co‐occurrence probability (*ψ*_AB_), a “Species Interaction Factor”, or *φ*, can also be obtained (MacKenzie et al., 2004; Richmond, Hines, & Beissinger, 2010). For species *A* and *B*, *φ* is defined as:

$$\varphi =\frac{\psi_{\mathrm{AB}}}{\psi_{A}\cdot \psi_{B}};$$

where *ψ_A_* and *ψ_B_* are the occupancy probabilities of species A and B, and *ψ_AB_* represents the co‐occurrence probability of species A and B. Under the null hypothesis, *φ* = 1, species populations exist independently and the pattern and frequency of species co‐occurrence are assumed to be random. If *φ* > 1, species co‐occur more frequently than expected from chance; likewise, *φ* < 1 indicates species co‐occur less frequently than chance. Using the same modeling procedure as with single‐species occupancy models, two‐species candidate models were fitted within the maximum‐likelihood framework provided by program PRESENCE (v. 11.7) under a ψ_Ba_‐parameterization (Richmond et al., 2010) and ranked using AIC. Predictions were obtained from the highest‐ranking models.

## 3 RESULTS

Repeated surveys of woodland salamanders at LCW resulted in the capture of 55 *P. richmondi* and 46 *P. kentucki*. *Plethodon richmondi* were detected at 25 of the total 40 sites surveyed (naïve proportion of area occupied [POA] = 0.63), and *P. kentucki* were detected at 26/40 sites (POA = 0.65).

### 3.1 Detection, Availability, and Temporary Emigration

**Figure 1:**
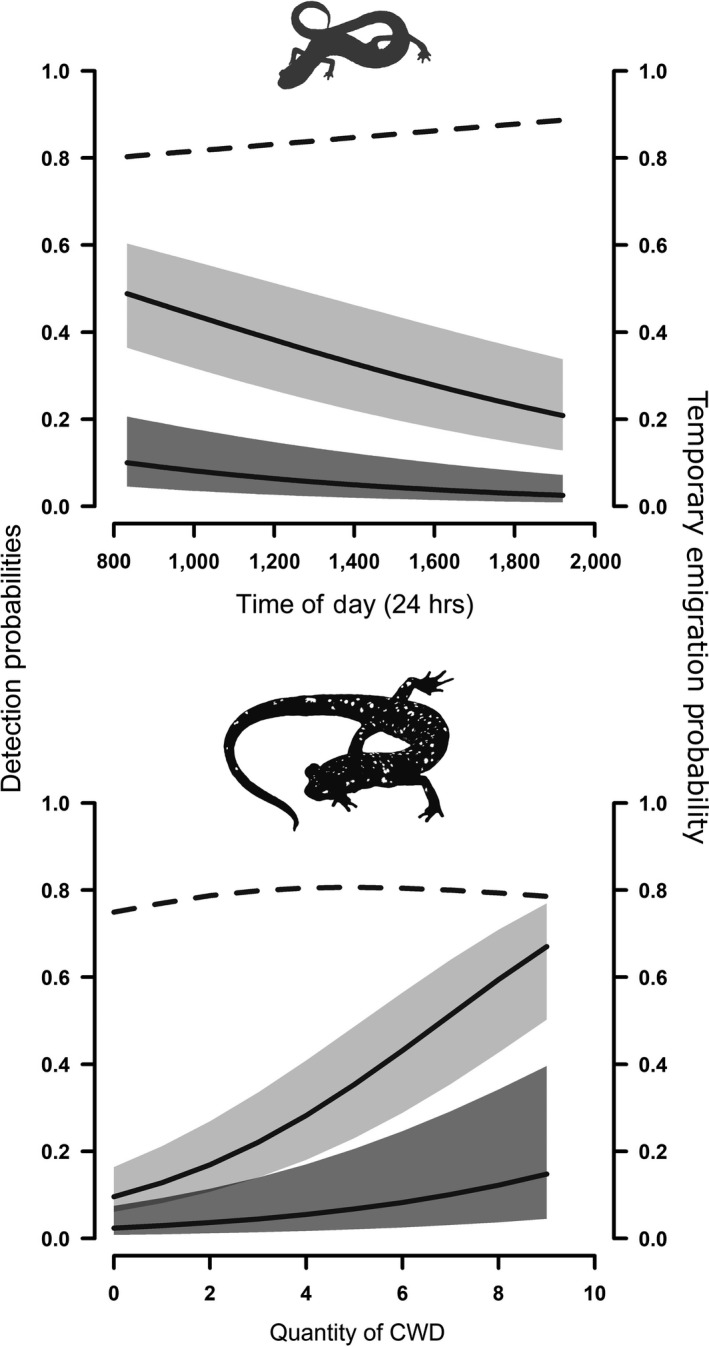
Estimates of Southern Ravine Salamander (*Plethodon richmondi*; top) and Cumberland Plateau Salamander (*P. kentucki*; bottom) temporary emigration probability (dotted lines) and two components of detection: conditional capture probability (light gray 95% CI) and effective detection probability (dark gray 95% CI) in relation to time of survey (top) and quantity of fallen CWD (coarse woody debris; bottom) in an old‐growth forest at Lilley Cornett Woods Appalachian Ecological Research Station, Letcher County, Kentucky, USA in Fall 2016

The conditional capture probabilities of *P. richmondi* and *P. kentucki* were moderately low (${\hat{\overset{¯}{p}}}_{\psi }$ = 0.36 and 0.24), while effective detection probabilities were much lower (${\hat{\overset{¯}{p}}}_{\lambda }$ = 0.06 and 0.05; Table 2). For *P. richmondi*, time of day (“TOD”) in which the survey occurred was the most important covariate for estimating ${\hat{\overset{¯}{p}}}_{\psi }$ (${\hat{\overset{¯}{\beta }}}_{p\psi }$ = −0.42 [95% unconditional CI: −0.83, −0.01]) and ${\hat{\overset{¯}{p}}}_{\lambda }$ (${\hat{\overset{¯}{\beta }}}_{p\lambda }$ = −0.43 [−0.75, −0.12]), and both components of detection decreased gradually from morning until evening (Figure 1). Availability of coarse woody debris (“CWD”) was the most important covariate in explaining both detectability parameters of *P. kentucki* (${\hat{\overset{¯}{\beta }}}_{p\psi }$ = 0.74 [0.34, 1.15], ${\hat{\overset{¯}{\beta }}}_{p\lambda }$ = 0.53 [0.21, 0.84]; Table 2). The conditional capture probability of *P. kentucki *increased sharply with rising quantities of fallen CWD, while the effective detection probability increased only gradually (Figure 1).

**Table 2:** Model‐averaged estimates of site and sampling parameters from hierarchical models of Southern Ravine Salamander (*Plethodon richmondi*) and Cumberland Plateau Salamander (*P. kentucki*)

Parameter	*P. richmondi*	*P. kentucki*
	95% CI	95% CI
Site		
Occupancy Probability,	0.74 (0.35, 0.89)	0.94 (0.12, 1.00)
Estimated Density (N/m)	0.06 (0.02, 0.15)	0.06 (0.02, 0.20)
Sampling		
Conditional Capture Probability, ${\hat{\overset{¯}{p}}}_{\psi }$	0.36 (0.25, 0.49)	0.24 (0.16, 0.35)
Effective Detection Probability, ${\hat{\overset{¯}{p}}}_{\lambda }$	0.06 (0.02, 0.14)	0.05 (0.02, 0.15)
Availability^a^	0.16	0.21
Emigration Probability^b^	0.84	0.79

Note

Data were collected from repeated (*N* = 4) surveys in 2016 at Lilley Cornett Woods Appalachian Ecological Research Station (Letcher Co., Kentucky, USA). Estimates are averages of *N* = 40 sites (95% CI).

a Defined as: 1—(Effective Detection Probability/Conditional Capture Probability).

b Defined as: 1—(Availability).

The probability that salamanders were alive, on the soil surface, and available for surveys (Availability probability) was low due to frequent vertical temporary emigration into the soil by both *P. richmondi* and *P. kentucki* (Table 2). The probability of temporary emigration by *P. richmondi* varied slightly with time of day, moderately increasing, linearly, from morning until evening. The temporary emigration probability of *P. kentucki* did not vary substantially, but exhibited a hump‐shaped relationship with the abundance of coarse woody debris (Figure 1).

### 3.2 Occupancy

**Figure 2:**
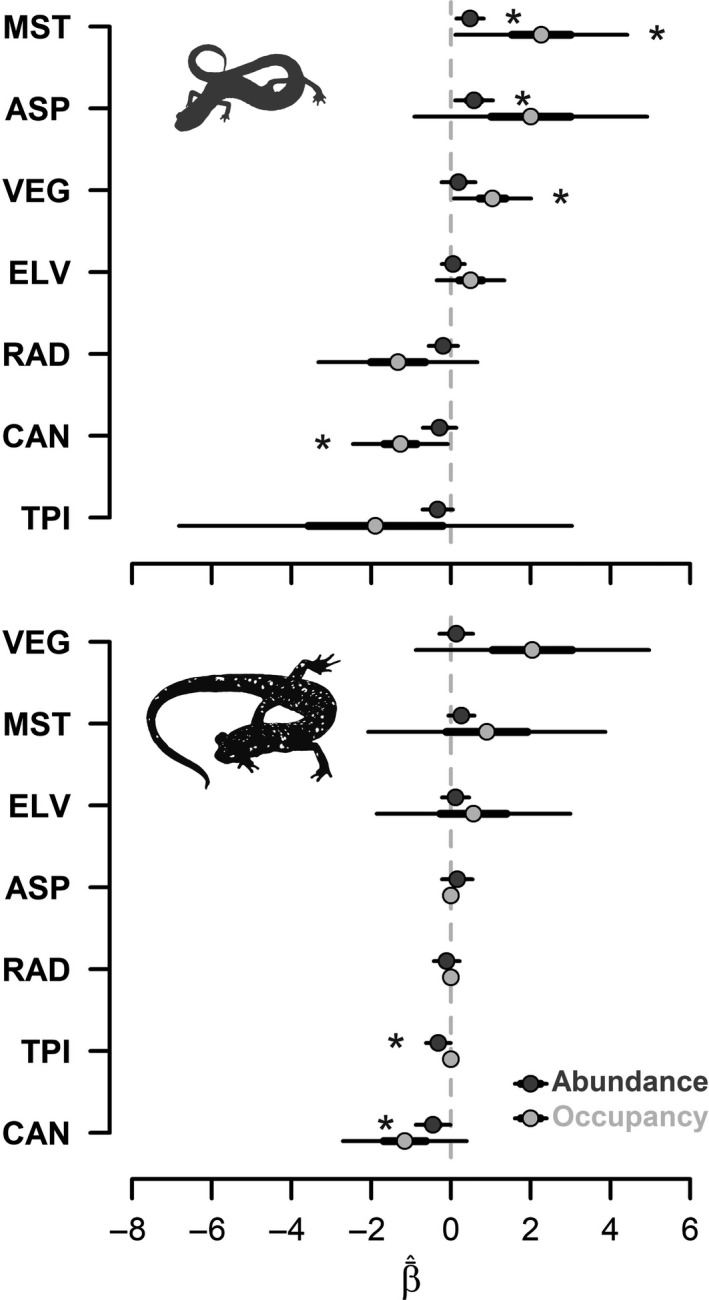
Model‐averaged estimates of effect sizes, $\hat{\overset{¯}{\beta }}$, of covariates used in occupancy (*ψ*, light gray) and N‐mixture models (*λ*, dark gray) of Southern Ravine Salamander (*Plethodon richmondi*; top) and Cumberland Plateau Salamander (*P. kentucki*; bottom) surveyed at Lilley Cornett Woods Appalachian Ecological Research Station, Letcher County, Kentucky, USA in Fall 2016. Thick lines around $\hat{\overset{¯}{\beta }}$ estimates represent a 50% unconditional confidence interval (CI), and thin lines represent a 95% CI. Asterisks represent $\hat{\overset{¯}{\beta }}$ estimates with 95% CI not containing “0.” “VEG” = Normalized Difference Vegetation Index, “ASP” = Beers‐transformed aspect, “TPI” = Topographic Position Index, “ELV” = Digital Elevation Model, “RAD” = direct solar radiation, “MST” = volumetric soil moisture, “CAN” = percent canopy openness. See Table 1 for a description of covariates. Covariates with $\hat{\overset{¯}{\beta }}$ values centered at zero were not estimated due to non‐convergent models or model instability and are therefore represented as having “zero” effect sizes

**Figure 3:**
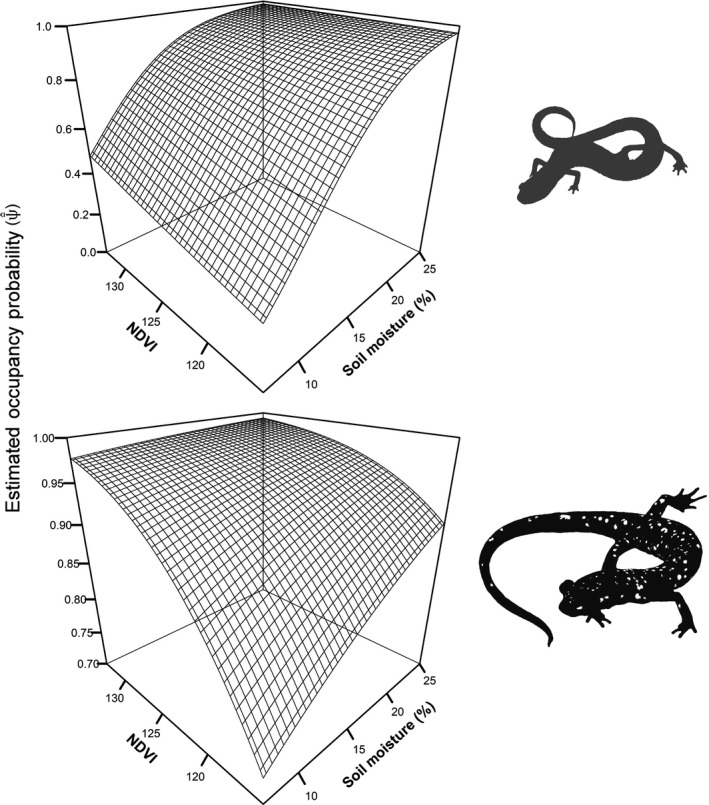
Occupancy probability ($\hat{\overset{¯}{\psi }}$) of Southern Ravine Salamander (*Plethodon richmondi*; top) and Cumberland Plateau Salamander (*P. kentucki*; bottom) in relation to NDVI (Normalized Difference Vegetation Index), a measure of vegetative cover, and percent soil moisture in an old‐growth forest at Lilley Cornett Woods Appalachian Ecological Research Station, Letcher County, Kentucky, USA in Fall 2016

*Plethodon richmondi* was predicted to have a moderately restricted distribution within LCW, with a model‐averaged occupancy estimate, $\hat{\overset{¯}{\psi }}$, of 0.738 (95% CI: 0.35, 0.89). Comparatively, *P*. *kentucki* was likely distributed more ubiquitously ($\hat{\overset{¯}{\psi }}$ = 0.947 [0.11, 1.0]; Table 2), although large confidence intervals provide considerable uncertainty in our assessment. Percent soil moisture (“MST”), NDVI (“VEG”), and canopy openness (“CAN”) were all important covariates in estimating occupancy of *P. richmondi* (Figure 2). Like *P. richmondi, P. kentucki* occupancy was also correlated with percent soil moisture and NDVI (Figure 3), but the directions of the covariates’ effects were heterogeneous (Figure 2). The remaining covariates included in models of occupancy produced heterogeneous effects and were therefore not considered to be reliable predictors of woodland salamander distributions in LCW.

### 3.3 Co‐occurrence

**Figure 4:**
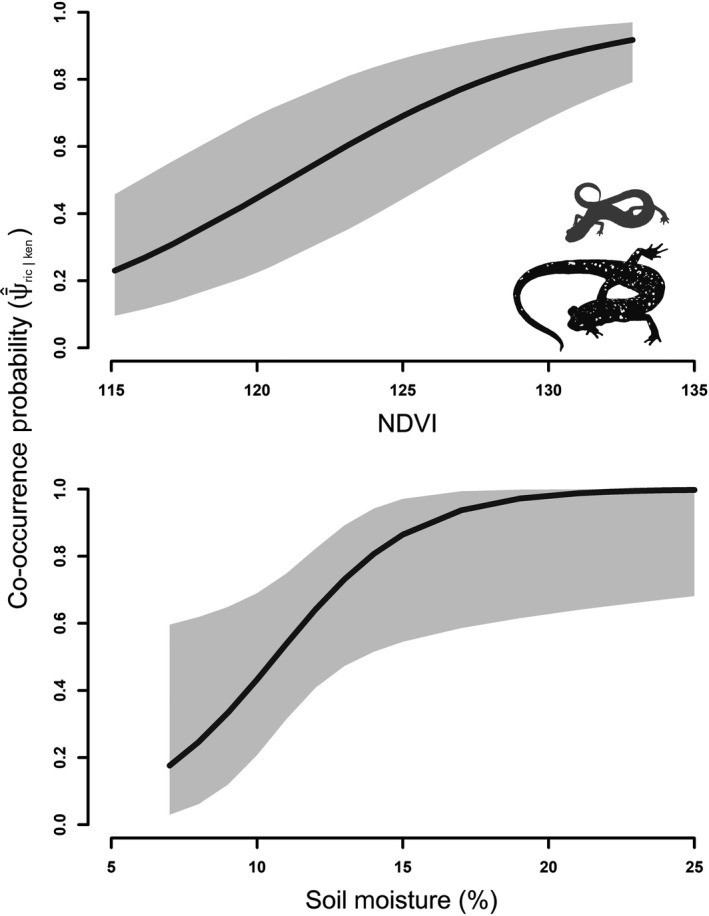
Co‐occurrence of Southern Ravine Salamander (*Plethodon richmondi*) and Cumberland Plateau Salamander (*P. kentucki*) in relation to NDVI (Normalized Difference Vegetation Index), a measure of vegetative cover, and percent soil moisture in an old‐growth forest at Lilley Cornett Woods Appalachian Ecological Research Station, Letcher County, Kentucky, USA in Fall 2016

The overall probability of *P. richmondi* co‐occurring with *P. kentucki*, ${\hat{\overset{¯}{\psi }}}_{ric|ken}$ was 0.72 (95% CI: 0.53, 0.86). Models of co‐occurrence featuring covariates that represent environmental gradients were better at predicting patterns of co‐occurrence (cumulative Akaike model weight [Σ*ω_ij_*] = 0.971) than null models (Σ*ω_ij_* = 0.029). Co‐occurrence probabilities were positively influenced by percent soil moisture and NDVI (Figure 4). The relationship of ${\hat{\overset{¯}{\psi }}}_{ric|ken}$ with NDVI was nearly linear, with a gradual positive slope. Co‐occurrence exhibited a steep positive slope where percent soil moisture <15%, plateauing at approximately 20%. These results provide evidence that species co‐occurrence patterns are nonrandom and vary along natural environmental gradients. However, the Species Interaction Factor, or *φ*, of *P. richmondi *and *P. kentucki* was equal to 1 ($\hat{\varphi }$ = 1.00; 95% CI = 0.984, 1.016), providing no evidence that competition affects co‐occurrence of *P. kentucki* and *P. richmondi* or that their distributions are spatially segregated.

### 3.4 Density

Density estimates obtained from N‐mixture models were substantially greater than counts uncorrected for imperfect detection, such that counts only represented 1.43%–7.22% (interquartile range) of the total estimated density of each species. When extrapolated to the total extent of the study area (44.25 ha), densities of *P. richmondi *and *P. kentucki* were estimated at 26,570 (95% CI: 10,895, 66,897) and 26,848 (95% CI: 8,552, 91,098), respectively.

**Figure 5:**
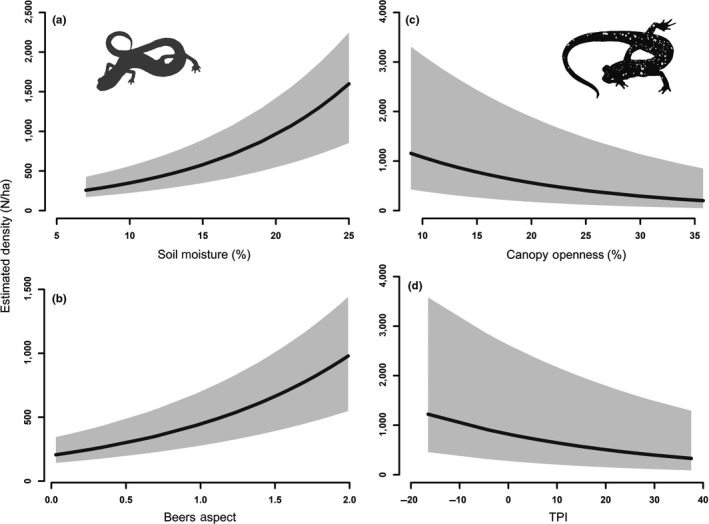
Estimated density (extrapolated to 1 ha) of Southern Ravine Salamander (*Plethodon richmondi*; panels A and B) in relation to percent soil moisture and Beers Aspect, a linearized measure of aspect (range: 0 [SW] – 2 [NE]); and Cumberland Plateau Salamander (*P. kentucki*; panels C and D) in relation to percent canopy closure and TPI (Topographic Position Index), a measure of landscape convexity, in an old‐growth forest at Lilley Cornett Woods Appalachian Ecological Research Station, Letcher County, Kentucky, USA in Fall 2016

Percent soil moisture (“MST”) and Beers‐transformed aspect (“ASP”) were the most important covariates when estimating density of *P. richmondi* (Figures 2 and 5). *Plethodon richmondi *density exhibited marked, positive curvilinear responses to percent soil moisture and aspect. *Plethodon kentucki* density was influenced most by Topographic Position Index (“TPI”) and percent canopy openness (“CAN”; Figure 2). The density of *P. kentucki* exhibited gradually dampened negative responses to both Topographic Position Index and percent canopy openness, with inflated upper limits (Figure 5).

## 4 DISCUSSION

We found that natural environmental gradients created by dynamic ecosystem processes inherent in old‐growth forest influence the fine‐scale distribution, co‐occurrence, and density of *P. richmondi* and *P. kentucki*. Species‐specific responses to gradients of soil moisture and temperature, solar exposure from canopy structure, and slope position likely reflect physiological restraints associated with desiccation vulnerability and thermal avoidance. Although patterns in co‐occurrence of *P. richmondi* and *P. kentucki* do vary along gradients of canopy density and soil moisture, little evidence was found to support the hypothesis that populations of woodland salamanders experience interspecific competition.

### 4.1 Plethodon richmondi

*Plethodon richmondi *density in LCW was positively related to forest soil moisture and reduced solar radiation due to slope face, and their fine‐scale distribution (i.e., occupancy) was restricted to mesic forest stands with minimal canopy disturbance. In LCW, canopy disturbance can be caused by natural, endogenous processes, such as (a) windthrow, which results either in mechanical removal of leaves and branches, or, in rare circumstances, complete root upheaval and (b) senescence or indirect damage from adjacent fallen trees, resulting in minor canopy disturbance. However, exogenous processes, such as the arrival of Hemlock Woolly Adelgid (*Adelges tsugae*), an invasive pest to Hemlock trees in eastern deciduous forests, have caused accelerated mortality of eastern Hemlock (*Tsuga canadensis*) in LCW. Tree mortality associated with *A. tsugae* is predicted to result in declines of black‐throated green warbler (*Setophaga virens*) in LCW and surrounding Appalachian forests in southeast Kentucky (Brown & Weinkam, 2014). Although *T. canadensis* stands in LCW are currently being treated for *A. tsugae*, tree mortality associated with this pest is presumed to continue. It follows that through alterations to canopy characteristics, *A. tsugae*, and other invasive pests in LCW (e.g., emerald ash borer,* Agrilus planipennis*) could negatively impact *Plethodon s*alamanders, which lack the vagility to evacuate habitats that have undergone dramatic transformation (Welsh & Droege, 2001). Further research into the mechanisms responsible for canopy loss in LCW may provide a more meaningful interpretation of *P. richmondi *occupancy and density dynamics. Future surveys and analyses should incorporate data pertaining to tree age, diameter, canopy density, and prevalence of pest‐related damage.

### 4.2 Plethodon kentucki

Density of *P. kentucki* was negatively impacted by the presence of canopy disturbance on exposed slope faces. Furthermore, canopy disturbance influenced the density of *P. kentucki* with a greater magnitude than soil moisture and aspect—gradients which both bolstered densities of *P. richmondi*. Perhaps the response of *P. kentucki *density to altered canopy structure was so great because canopy disturbance directly erodes environmental conditions which typically promote local *Plethodon *salamander population viability (e.g., moist soil and low solar exposure; Ford, Menzel, and Odom (), Peterman & Semlitsch, 2013; Semlitsch et al., 2014).

Factors affecting local density of *P. kentucki* did not necessarily affect their distribution. Specifically, canopy disturbance and landscape convexity (exposure) were found to be key determinants of density at a given site, but did not necessarily influence the likelihood of that site being occupied. These results suggest that within LCW *P. kentucki *respond with greater consequence to environmental processes which govern population size (i.e., productivity, recruitment) than those which perhaps govern their occurrence (i.e., colonization, extinction).

It is possible that aspects of environmental gradients assessed in this study were not important in explaining population dynamics of *P. kentucki* or *P. richmondi* due to the coarse scale with which they were evaluated. For instance, environmental variables and salamander counts were aggregated to the extent of the sampling area (800 m^2^), a scale which could obscure relevant information about the relationship of salamanders with micro‐scale variation in environmental conditions.

### 4.3 Co‐occurrence

The degree of overlap in the fine‐scale distributions of *P. richmondi* and *P. kentucki* within LCW corresponded strongly with natural environmental gradients. The probability of *P. richmondi* and *P. kentucki* co‐occurring in a given forest stand at LCW was positively correlated with soil moisture and canopy density. More specifically, co‐occurrence was more common between *P. richmondi *and *P. kentucki* in mesic habitats, where stress associated with desiccation avoidance and thermoregulation is minimal; co‐occurrence was much less common in xeric habitats with dry, clay‐dominated soils, and sparse canopy coverage, where physical stress is likely most apparent. However, there is no evidence to suggest that the occurrence of one species is influenced by the presence of another; their populations likely occur independently. There is also little evidence to suggest that microhabitat usage (i.e., coarse woody debris, rocky cover, leaf litter) differs between these species (Baecher & Richter unpubl. data). Perhaps observed patterns in co‐occurrence of *P. richmondi *and *P. kentucki* are artifacts of the individual occurrence pattern of *P. richmondi*, given *P. kentucki *was so ubiquitously distributed.

If *P. richmondi* and *P. kentucki *populations do in fact experience interspecific competition and are not independent, it is possible that the methods applied in this study were insufficient to detect such phenomena. For instance, if *Plethodon *salamanders ameliorate competitive pressure through spatial reorganization of territories, which can occur on scales equivalent to the cumulative area of the focal individuals’ home ranges (Marvin, 1998), it is possible that the spatial scale of this study was too coarse to quantify such fine‐scale interactions. Another potential explanation of the observed patterns of co‐occurrence may be related to mating behavior of *P. kentucki*. Marvin (1998) found that populations of *P. kentucki *in this region exhibit territoriality associated with mate pairing. In southeast Kentucky, the breeding period of *P. kentucki* begins late June to mid‐August and lasts until mid‐to‐late October (Baecher pers. obs., Marvin & Hutchison, 1996). Although unrelated to interspecific competition, it is possible that territoriality associated with *P. kentucki* breeding behavior was not observed during the timeframe of this study (15 October 2016 to 13 November 2016).

### 4.4 Detection, Availability, and Temporary Emigration

Unless all individuals in a population are available for capture during a survey (availability = 1), it is important to distinguish between conditional capture probability (probability of capturing an animal given availability = 1) and effective detection probability (probability of capturing an animal given availability ≤1). Given that *Plethodon *are known to migrate between surface and subsurface refugia frequently (Bailey, Simons, & Pollock, 2004), their availability—the probability of an individual being alive and present on the soil surface during a survey—should be much <1 (availability = 1 – [temporary emigration]; O'Donnell et al., 2015), and therefore, estimates of effective detection probability should be much less than that of the conditional capture probability. Corroborating the assertion that surface inactivity confounds studies of *Plethodon *salamanders (Bailey et al., 2004; O'Donnell et al., 2015), this study showed that more than half of the individuals in populations of *P. richmondi *and *P. kentucki* had emigrated into subterranean refugia and were unavailable for surveying.

## 5 CONCLUSIONS

This study found that the pattern of distribution and the abundance of woodland salamanders throughout the landscape can be nonrandom. Given that the nature of woodland salamanders’ effects on forest floor dynamics (e.g., detrital food webs, organic material retention) can change due to variation in environmental conditions (Walton, 2005, 2013), it is likely that the spatial extent of woodland salamander's influence on the ecosystem is nonrandom and varies dramatically across natural environmental gradients (Semlitsch et al., 2014). Thus, the role that woodland salamanders play in the maintenance of forest health, biodiversity, and ecosystem services (Davic & Welsh, 2004) is likely contingent upon the inherent inhabitability of the system. Therefore, regions within a forest that support large populations of woodland salamanders, such as those highlighted in this study—mesic forest stands on north‐to‐east facing slopes with dense canopy—may provide enhanced ecosystem services and support stability in the total forest ecosystem (Davic & Welsh, 2004). This study took place in a stable old‐growth forest (Martin, 1975), virtually undisturbed by human activity (with the exception of light recreation from guided hiking). Because woodland salamanders in this study exhibited such marked responses to natural disturbances associated with forested ecosystems (e.g., isolated canopy perforation and soil desiccation due to solar exposure), population‐level responses to nonnatural disturbances (e.g., timber harvest and residential/commercial development) are hypothesized to be much more substantial.

## AUTHORS’ CONTRIBUTIONS

JAB and SCR conceived the ideas and designed the methodology; JAB collected and analyzed the data; JAB and SCR wrote the manuscript.

## DATA ACCESSIBILITY

Materials to reproduce analyses are freely available for download by visiting the following repositories. Raw data: https://doi.org/10.6073/pasta/d834e12ff12dc23d9319fa9f73e40306;R code: http://doi.org/10.5281/zenodo.1313705.
