# Supplementary material for: Environmental gradients in old‐growth Appalachian forest predict fine‐scale distribution, co‐occurrence, and density of woodland salamanders
Source: Ecol Evol. 2018 Dec 4;8(24):12940–52. doi: 10.1002/ece3.4736 (PMC6308888; doi:10.1002/ece3.4736)
Supplement: Supplementary file 1 [file ECE3-8-12940-s001.docx]

Appendix 1: AIC table of single season abundance (*N*-mixture) models of counts from repeated surveys (N=4) of *Plethodon richmondi* at Lilley Cornett Woods Appalachian Ecological Research Station (Letcher Co., KY) in Fall 2016. Detection models were parameterized with sampling covariates, and the state parameter, λ, is held constant. Abundance models are parameterized with sampling covariates and site covariates. A Poisson distribution was used for all models.

| **Parameterization** | | | | **K** | **ΔAICc** | **ω**_i_ |
| --- | --- | --- | --- | --- | --- | --- |
| **Detection** | |  |  |  |  |  |
|  | p(CWD+TOD) | , | λ(.) | 4 | 0.00 | 0.49 |
|  | p(TOD) | , | λ(.) | 3 | 1.68 | 0.21 |
|  | p(TOD+DAY) | , | λ(.) | 4 | 3.36 | 0.09 |
|  | p(ROC+TOD) | , | λ(.) | 4 | 3.64 | 0.08 |
|  | p(CWD) | , | λ(.) | 3 | 5.25 | 0.04 |
|  | p(ROC+LUX+DAY+CWD+TOD+LLD) | , | λ(.) | 8 | 6.61 | 0.02 |
|  | p(CWD+LUX) | , | λ(.) | 4 | 6.88 | 0.02 |
|  | p(CWD+DAY) | , | λ(.) | 4 | 6.95 | 0.02 |
|  | p(ROC+CWD) | , | λ(.) | 4 | 7.20 | 0.01 |
|  | p(.) | , | λ(.) | 2 | 7.51 | 0.01 |
|  | p(LUX) | , | λ(.) | 3 | 8.85 | 0.01 |
|  | p(LLD) | , | λ(.) | 3 | 9.36 | 0.00 |
|  | p(DAY) | , | λ(.) | 3 | 9.44 | 0.00 |
|  | p(ROC) | , | λ(.) | 3 | 9.49 | 0.00 |
| **Abundance** | |  |  |  |  |  |
|  | p(TOD) | , | λ(TPI+ASP) | 5 | 0.00 | 0.25 |
|  | p(TOD) | , | λ(ASP+MST) | 5 | 0.52 | 0.20 |
|  | p(TOD) | , | λ(MST) | 4 | 2.01 | 0.09 |
|  | p(TOD) | , | λ(RAD+MST) | 5 | 2.31 | 0.08 |
|  | p(TOD) | , | λ(CAN+ASP) | 5 | 2.64 | 0.07 |
|  | p(TOD) | , | λ(CAN+MST) | 5 | 2.85 | 0.06 |
|  | p(TOD) | , | λ(VEG+MST) | 5 | 2.95 | 0.06 |
|  | p(TOD) | , | λ(ELV+MST) | 5 | 3.62 | 0.04 |
|  | p(TOD) | , | λ(ASP) | 4 | 3.82 | 0.04 |
|  | p(TOD) | , | λ(TPI+MST) | 5 | 3.98 | 0.04 |
|  | p(.) | , | λ(MST) | 3 | 5.22 | 0.02 |
|  | p(TOD) | , | λ(VEG+ASP) | 5 | 5.54 | 0.02 |
|  | p(TOD) | , | λ(ELV+ASP) | 5 | 5.75 | 0.01 |
|  | p(TOD) | , | λ(RAD+ASP) | 5 | 5.79 | 0.01 |
|  | p(TOD) | , | λ(CAN+MST+ELV+ASP+VEG+RAD+TPI) | 10 | 8.17 | 0.00 |
|  | p(TOD) | , | λ(RAD) | 4 | 9.52 | 0.00 |
|  | p(TOD) | , | λ(CAN) | 4 | 9.73 | 0.00 |
|  | p(.) | , | λ(ASP) | 3 | 10.64 | 0.00 |
|  | p(TOD) | , | λ(VEG) | 4 | 11.92 | 0.00 |
|  | p(TOD) | , | λ(TPI) | 4 | 13.25 | 0.00 |
|  | p(.) | , | λ(RAD) | 3 | 15.09 | 0.00 |
|  | p(.) | , | λ(VEG) | 3 | 17.12 | 0.00 |
|  | p(.) | , | λ(CAN) | 3 | 17.19 | 0.00 |
|  | p(.) | , | λ(TPI) | 3 | 17.98 | 0.00 |
|  | p(TOD) | , | λ(.) | 3 | 18.19 | 0.00 |
|  | p(TOD) | , | λ(ELV) | 4 | 20.03 | 0.00 |
|  | p(.) | , | λ(.) | 2 | 24.02 | 0.00 |
|  | p(.) | , | λ(ELV) | 3 | 25.85 | 0.00 |
| K = # parameters, AIC = Akaike's Information Criteria, ΔAIC = AIC_i_ - AIC_top model_, ω_i_ = model weight | | | | | | |

Appendix 2: AIC table of single season occupancy models of presence/absence data collected from repeated surveys (N=4) of *P. richmondi* at Lilley Cornett Woods Appalachian Ecological Research Station (Letcher Co., KY) in Fall 2016. Detection models are parameterized with sampling covariates, and the state parameter, ψ, is held constant. Occupancy models are parameterized with sampling covariates and site covariates.

| **Parameterization** | | | | **K** | **ΔAICc** | **ω**_i_ |
| --- | --- | --- | --- | --- | --- | --- |
| **Detection** | |  |  |  |  |  |
|  | p(TOD) | , | ψ(.) | 3 | 0.00 | 0.23 |
|  | p(ROC+TOD) | , | ψ(.) | 4 | 0.28 | 0.20 |
|  | p(CWD+TOD) | , | ψ(.) | 4 | 1.38 | 0.12 |
|  | p(TOD+DAY) | , | ψ(.) | 4 | 1.71 | 0.10 |
|  | p(.) | , | ψ(.) | 2 | 1.99 | 0.09 |
|  | p(ROC) | , | ψ(.) | 3 | 2.99 | 0.05 |
|  | p(CWD) | , | ψ(.) | 3 | 3.12 | 0.05 |
|  | p(LUX) | , | ψ(.) | 3 | 3.77 | 0.04 |
|  | p(LLD) | , | ψ(.) | 3 | 3.82 | 0.03 |
|  | p(DAY) | , | ψ(.) | 3 | 3.95 | 0.03 |
|  | p(ROC+CWD) | , | ψ(.) | 4 | 4.59 | 0.02 |
|  | p(CWD+LUX) | , | ψ(.) | 4 | 4.96 | 0.02 |
|  | p(CWD+DAY) | , | ψ(.) | 4 | 5.00 | 0.02 |
|  | p(ROC+LUX+DAY+CWD+TOD+LLD) | , | ψ(.) | 8 | 8.02 | 0.00 |
| **Occupancy** | |  |  |  |  |  |
|  | p(TOD) | , | ψ(MST) | 4 | 0.00 | 0.38 |
|  | p(TOD) | , | ψ(ASP) | 4 | 1.30 | 0.20 |
|  | p(.) | , | ψ(MST) | 3 | 1.85 | 0.15 |
|  | p(TOD) | , | ψ(CAN) | 4 | 3.04 | 0.08 |
|  | p(TOD) | , | ψ(VEG) | 4 | 4.37 | 0.04 |
|  | p(TOD) | , | ψ(RAD) | 4 | 4.90 | 0.03 |
|  | p(.) | , | ψ(ASP) | 3 | 5.00 | 0.03 |
|  | p(.) | , | ψ(CAN) | 3 | 5.54 | 0.02 |
|  | p(.) | , | ψ(VEG) | 3 | 6.70 | 0.01 |
|  | p(.) | , | ψ(RAD) | 3 | 7.36 | 0.01 |
|  | p(TOD) | , | ψ(.) | 3 | 7.68 | 0.01 |
|  | p(TOD) | , | ψ(TPI) | 4 | 7.89 | 0.01 |
|  | p(TOD) | , | ψ(ELV) | 4 | 8.39 | 0.01 |
|  | p(.) | , | ψ(TPI) | 3 | 9.25 | 0.00 |
|  | p(.) | , | ψ(.) | 2 | 9.67 | 0.00 |
|  | p(.) | , | ψ(ELV) | 3 | 10.36 | 0.00 |
| K = # parameters, AIC = Akaike's Information Criteria, ΔAIC = AIC_i_ - AIC_top model_, ω_i_ = model weight | | | | | | |

Appendix 3: AIC table of single season abundance (*N*-mixture) models of counts from repeated surveys (N=4) of *Plethodon kentucki* at Lilley Cornett Woods Appalachian Ecological Research Station (Letcher Co., KY) in Fall 2016. Detection models were parameterized with sampling covariates, and the state parameter, λ, is held constant. Abundance models are parameterized with sampling covariates and site covariates. A Poisson distribution was used for all models.

| **Parameterization** | | | | **K** | **ΔAICc** | **ω**_i_ |
| --- | --- | --- | --- | --- | --- | --- |
| **Detection** | |  |  |  |  |  |
|  | p(ROC+CWD) | , | λ(.) | 4 | 0.00 | 0.67 |
|  | p(CWD) | , | λ(.) | 3 | 3.14 | 0.14 |
|  | p(CWD+TOD) | , | λ(.) | 4 | 4.89 | 0.06 |
|  | p(CWD+DAY) | , | λ(.) | 4 | 5.01 | 0.05 |
|  | p(CWD+LUX) | , | λ(.) | 4 | 5.12 | 0.05 |
|  | p(ROC+LUX+DAY+CWD+TOD+LLD) | , | λ(.) | 8 | 6.93 | 0.02 |
|  | p(ROC) | , | λ(.) | 3 | 9.77 | 0.01 |
|  | p(ROC+TOD) | , | λ(.) | 4 | 11.61 | 0.00 |
|  | p(.) | , | λ(.) | 2 | 12.35 | 0.00 |
|  | p(DAY) | , | λ(.) | 3 | 13.62 | 0.00 |
|  | p(TOD) | , | λ(.) | 3 | 14.26 | 0.00 |
|  | p(LLD) | , | λ(.) | 3 | 14.28 | 0.00 |
|  | p(LUX) | , | λ(.) | 3 | 14.29 | 0.00 |
|  | p(TOD+DAY) | , | λ(.) | 4 | 15.62 | 0.00 |
| **Abundance** | |  |  |  |  |  |
|  | p(CWD) | , | λ(CAN+TPI) | 5 | 0.00 | 0.48 |
|  | p(CWD) | , | λ(CAN) | 4 | 1.35 | 0.24 |
|  | p(CWD) | , | λ(TPI) | 4 | 1.94 | 0.18 |
|  | p(CWD) | , | λ(MST) | 4 | 5.33 | 0.03 |
|  | p(CWD) | , | λ(CAN+MST+ASP+ELV+VEG+TPI+RAD) | 10 | 6.65 | 0.02 |
|  | p(CWD) | , | λ(ASP) | 4 | 7.64 | 0.01 |
|  | p(CWD) | , | λ(VEG) | 4 | 7.64 | 0.01 |
|  | p(CWD) | , | λ(RAD) | 4 | 7.94 | 0.01 |
|  | p(CWD) | , | λ(ELV) | 4 | 7.99 | 0.01 |
|  | p(.) | , | λ(CAN) | 3 | 9.49 | 0.00 |
|  | p(.) | , | λ(TPI) | 3 | 10.91 | 0.00 |
|  | p(.) | , | λ(MST) | 3 | 12.67 | 0.00 |
|  | p(.) | , | λ(ASP) | 3 | 15.40 | 0.00 |
|  | p(.) | , | λ(VEG) | 3 | 15.50 | 0.00 |
|  | p(.) | , | λ(.) | 2 | 15.59 | 0.00 |
|  | p(.) | , | λ(ELV) | 3 | 16.76 | 0.00 |
|  | p(.) | , | λ(RAD) | 3 | 16.79 | 0.00 |
| K = # parameters, AIC = Akaike's Information Criteria, ΔAIC = AIC_i_ - AIC_top model_, ω_i_ = model weight | | | | | | |

Appendix 4: AIC table of single season occupancy models of presence/absence data collected from repeated surveys (N=4) of *P. kentucki* at Lilley Cornett Woods Appalachian Ecological Research Station (Letcher Co., KY) in Fall 2016. Detection models are parameterized with sampling covariates, and the state parameter, ψ, is held constant. Occupancy models are parameterized with sampling covariates and site covariates.

| **Parameterization** | | | | **K** | **ΔAICc** | **ω**_i_ |
| --- | --- | --- | --- | --- | --- | --- |
| **Detection** | |  |  |  |  |  |
|  | p(ROC+CWD) | , | ψ(.) | 4 | 0.00 | 0.31 |
|  | p(CWD) | , | ψ(.) | 3 | 0.49 | 0.25 |
|  | p(CWD+TOD) | , | ψ(.) | 4 | 0.84 | 0.21 |
|  | p(CWD+DAY) | , | ψ(.) | 4 | 1.91 | 0.12 |
|  | p(CWD+LUX) | , | ψ(.) | 4 | 2.49 | 0.09 |
|  | p(ROC+LUX+DAY+CWD+TOD+LLD) | , | ψ(.) | 8 | 5.46 | 0.02 |
|  | p(ROC) | , | ψ(.) | 3 | 12.74 | 0.00 |
|  | p(.) | , | ψ(.) | 2 | 13.15 | 0.00 |
|  | p(DAY) | , | ψ(.) | 3 | 13.56 | 0.00 |
|  | p(ROC+TOD) | , | ψ(.) | 4 | 13.59 | 0.00 |
|  | p(TOD) | , | ψ(.) | 3 | 14.33 | 0.00 |
|  | p(LUX) | , | ψ(.) | 3 | 14.90 | 0.00 |
|  | p(LLD) | , | ψ(.) | 3 | 15.15 | 0.00 |
|  | p(TOD+DAY) | , | ψ(.) | 4 | 15.38 | 0.00 |
| **Occupancy** | |  |  |  |  |  |
|  | p(CWD) | , | ψ(.) | 3 | 0.00 | 0.36 |
|  | p(CWD) | , | ψ(VEG) | 4 | 0.50 | 0.28 |
|  | p(CWD) | , | ψ(CAN) | 4 | 0.94 | 0.22 |
|  | p(CWD) | , | ψ(MST) | 4 | 1.92 | 0.14 |
|  | p(.) | , | ψ(CAN) | 3 | 11.23 | 0.00 |
|  | p(.) | , | ψ(VEG) | 3 | 11.50 | 0.00 |
|  | p(.) | , | ψ(.) | 2 | 12.66 | 0.00 |
|  | p(.) | , | ψ(ELV) | 3 | 14.53 | 0.00 |
| K = # parameters, AIC = Akaike's Information Criteria, ΔAIC = AIC_i_ - AIC_top model_, ω_i_ = model weight | | | | | | |

Appendix 5: AIC table of single season two-species occupancy (co-occurrence) models of presence/absence data collected from repeated surveys (N=4) of *Plethodon richmondi and Plethodon kentucki* at Lilley Cornett Woods Appalachian Ecological Research Station (Letcher Co., KY) in Fall 2016. Detection models are parameterized with sampling covariates, and the state parameter, ψ, is held constant. Co-occurrence models are parameterized with sampling covariates and site covariates.

| **Parameterization** | | | | | **K** | **ΔAICc** | **ω**_i_ |
| --- | --- | --- | --- | --- | --- | --- | --- |
| **Detection** | |  |  |  |  |  |  |
|  | p'(CWD), p"(CWD), r'(CWD), r''(CWD), r'''(CWD) | , | ψ'(.), ψ''(.), ψ'''(.) |  | 8 | 0.00 | 0.71 |
|  | p'(CWD+TOD), p"(CWD+TOD), r'(CWD+TOD), r''(CWD+TOD), r'''(CWD+TOD) | , | ψ'(.), ψ''(.), ψ'''(.) |  | 9 | 1.99 | 0.26 |
|  | p'(.), p"(.), r'(.), r''(.), r'''(.) | , | ψ'(.), ψ''(.), ψ'''(.) |  | 10 | 7.55 | 0.02 |
|  | p'(TOD), p"(TOD), r'(TOD), r''(TOD), r'''(TOD) | , | ψ'(.), ψ''(.), ψ'''(.) |  | 9 | 9.40 | 0.01 |
| **C-occurrence** | |  |  |  |  |  |  |
|  | p'(CWD), p"(CWD), r'(CWD), r''(CWD), r'''(CWD) | , | ψ'(MST), ψ''(MST), ψ'''(MST) |  | 10 | 0.00 | 0.79 |
|  | p'(CWD), p"(CWD), r'(CWD), r''(CWD), r'''(CWD) | , | ψ'(VEG), ψ''(VEG), ψ'''(VEG) |  | 10 | 2.91 | 0.18 |
|  | p'(CWD), p"(CWD), r'(CWD), r''(CWD), r'''(CWD) | , | ψ'(.), ψ''(.), ψ'''(.) |  | 9 | 6.40 | 0.03 |
| K = # parameters, AIC = Akaike's Information Criteria, ΔAIC = AIC_i_ - AIC_top model_, ω_i_ = model weight | | | | | | | |
|  |  |  |  |  |  |  |  |
| **Parameterization explained** | | | | | | | |
| **Term** | **Definition** |  | **Symbology** | | | | |
| ψ' | Probability of occupancy for *P. kentucki* | | ψ*_ken_* | | | | |
| ψ'' | Probability of occupancy for *P. richmondi*, given *P. kentucki* is present | | ψ_ric \| ken†_ | | | | |
| ψ''' | Probability of occupancy for *P. richmondi*, given *P. kentucki* is absent | | ψ_ric \| ~~ken~~_ | | | | |
| p' | Probability of detection for *P. kentucki*, given *P. richmondi* is absent | | p_ken \| ~~ric~~_ | | | | |
| p'' | Probability of detection for *P. richmondi*, given *P. kentucki* is absent | | p_ric \| ~~ken~~_ | | | | |
| r' | Probability of detection for *P. kentucki*, given *P.* *richmondi* and *P. kentucki* are present | | p_ken \| ric+ken_ | | | | |
| r'' | Probability of detection for *P. richmondi*, given both species are present and *P. kentucki* is detected | | p_ric \| ric+ken x ken_ | | | | |
| r''' | Probability of detection for *P. richmondi*, given both species are present and *P. kentucki* is not detected | | p_ric \| ric+ken x ken_ | | | | |
